# Transcriptome Profiling of the Lungs Reveals Molecular Clock Genes Expression Changes after Chronic Exposure to Ambient Air Particles

**DOI:** 10.3390/ijerph14010090

**Published:** 2017-01-18

**Authors:** Pengcheng Song, Zhigang Li, Xiaoqian Li, Lixin Yang, Lulu Zhang, Nannan Li, Chen Guo, Shuyu Lu, Yongjie Wei

**Affiliations:** 1 College of Environmental Science and Engineering, Dong Hua University, Shanghai 201620, China; penches@163.com (P.S.); sisrlu@126.com (S.L.); 2 Laboratory of Environmental Criteria and Risk Assessment & Environmental Standards Institute, Chinese Research Academy of Environmental Sciences, Beijing 100012, China; lixiaoqian@craes.org.cn (X.L.); yanglx@craes.org.cn (L.Y.); zhang_lulu@yeah.net (L.Z.); linannqinian@163.com (N.L.); chenpreteen@sina.com (C.G.)

**Keywords:** ambient air particles, transcriptome, lung, rhythm, clock genes

## Abstract

The symptoms of asthma, breathlessness, insomnia, etc. all have relevance to pulmonary rhythmic disturbances. Epidemiology and toxicology studies have demonstrated that exposure to ambient air particles can result in pulmonary dysfunction. However, there are no data directly supporting a link between air pollution and circadian rhythm disorder. In the present study, we found that breathing highly polluted air resulted in changes of the molecular clock genes expression in lung by transcriptome profiling analyses in a rodent model. Compared to those exposed to filtered air, in both pregnant and offspring rats in the unfiltered group, key clock genes (*Per1*, *Per2*, *Per3*, *Rev-erbα* and *Dbp*) expression level decreased and *Bmal1* expression level increased. In both rat dams and their offspring, after continuous exposure to unfiltered air, we observed significant histologic evidence for both perivascular and peribronchial inflammation, increased tissue and systemic oxidative stress in the lungs. Our results suggest that chronic exposure to particulate matter can induce alterations of clock genes expression, which could be another important pathway for explaining the feedbacks of ambient particle exposure in addition to oxidative stress and inflammation.

## 1. Introduction

In human and other mammals, circadian rhythms are intrinsic oscillations with a period near 24 h, which are synchronized by some environmental cues, such as light and food [1,2,3]. The central pacemaker for controlling circadian rhythms is localized in the suprachiasmatic nucleus (SCN) in the ventral hypothalamus [1,4,5]. The brain and peripheral tissues, including lung tissue, also have autonomous circadian oscillators [4,6]. Moreover, the circadian clock governs many important behaviors and physiological processes, including feeding, sleeping/waking, hormone secretion, body temperature and metabolism [7]. Circadian rhythm can provide the time cues of activities and synchronize the metabolic reactions with anticipated cycles. It is reported that shift workers have higher risks of metabolic syndrome, including obesity, cardiovascular diseases, and diabetes [8], related to circadian rhythm disruption.

It is well known that exposure to ambient air particulate matters (PM) can induce oxidative stress and excessive pulmonary inflammation [9]. Chronic exposure to PM is inextricably linked to the metabolic syndrome [10]. Previous studies have revealed that interference with the circadian molecular clock function could impair the innate immune response, suggesting a crucial role of the circadian oscillator in maintaining normal immune responses [11]. The normal timing and amplitude were disrupted in the lungs of many patients with nocturnal asthma [12,13,14,15]. Patients with chronic obstructive pulmonary disease (COPD) also showed rhythmic variation, with symptoms of nocturnal breathlessness, insomnia, etc., which stem from altering the clock function in lung tissue [16,17,18]. As one of the pathogenic factors, PM can also lead to many lung diseases, such as asthma, COPD, pneumonia, bronchitis [19]. However, it is still not clear whether inhaled PM can alter the circadian clock function in the lungs.

Hence, we hypothesized that chronic exposure to PM can have an effect on the circadian functions in the lungs through regulating the circadian rhythm related genes by a certain pathway/mechanism. To address this hypothesis, we analyzed the expression levels of clock genes in the lung tissue of rats and tried to find an association between the circadian functions and the immune system.

## 2. Materials and Methods

### 2.1. Ethics

All animal experiments were conducted in compliance with the guidelines of ethical animal research. The study protocol was approved by the Medicine Animal Care and Use Committee at Peking University Health Science Center before commencement of experiments (ethical code LA2009268).

### 2.2. Exposure Protocol

As in our previous paper [10], two identically sized chambers, each ~1m^3^ in volume (1.2 × 0.8 × 1.2 m), were used in the present study. The chambers are divided into two layers. Each chamber could house a maximum of nine rat cages (four on the upper rack, and five on the lower rack). Only two rats were housed in each cage (the same as normal feeding conditions). This ensures no overcrowding and that they can move freely in the cages. These chambers were placed side by side for whole body inhalation exposure of rats in a clean-level air-conditioned room. The ambient air is ducted to the chambers though the pipes directly from outside (the real-world) by a suction blower and delivered to the chambers by the inlet duct on the top of the chambers. The only difference between the two chambers was the presence or absence of a high-efficiency particulate air (HEPA) filter placed in the inlet duct. The room was located in a heavy traffic area in Haidian District in Beijing. Particulate matter concentrations were measured using two instrument sets comprised of a single particle soot photometer and scanning mobility particle sizer and aerodynamic particle size, one for the chamber airstream and the other for ambient atmosphere. The removal efficiency of the HEPA filter in the chamber was 98.99% ± 0.86% for particles larger than 2.5 mm in diameter and 70.61% ± 19.34% for PM_2.5_. PM_2.5_ concentrations inside the chamber equipped with the HEPA filter were estimated using outdoor PM_2.5_ concentrations and the filter removal efficiency for PM_2.5_. The temperature inside these chambers was controlled at 24 ± 1 °C.

We started our experiments with pregnant Sprague Dawley rats. All the rats were kept in an animal care facility before the experiments commenced. We conducted the first set of experiments using 30 pregnant Sprague Dawley rats that were 12 weeks old. On gestation day 4, rats were randomized into two groups: 18 were placed in the unfiltered chamber, and the remaining 12 in the filtered chamber. Rats lived in these chambers naturally under a 12 h light/12 h dark cycle and were fed a normal chow diet. On gestation days 5–18, (28 December 2009, to 10 January 2010), PM_2.5_ concentrations were 73.5 ± 61.3 μg/m^3^ in the unfiltered chamber and 19.8 ± 9.0 μg/m^3^ in the filtered chamber. We further had the pups of rat dams prenatally exposed to unfiltered air stay in the unfiltered chamber, and the male pups prenatally exposed to filtered air stayed in the filtered chamber continuously until they were analyzed at 8 weeks of age.

To confirm some of the previous findings, we conducted a 2nd set of experiments using 16 pregnant SD rats at 12 weeks of age (10 in the unfiltered air group and six in the filtered air group). The experiments started at gestation day 1 and lasted for 19 days. Pups of these rats were not further studied. The exposure protocol was the same as the first set of experiment. The total exposure period was 19 days (12–31 March 2010), PM_2.5_ concentrations were 64.6 ± 72.7 μg/m^3^ and 16.7 ± 9.6 μg/m^3^ in the unfiltered and the filtered chamber, respectively. After PM_2.5_ exposure, rats were sacrificed at ZT4 (where ZT0 is defined as the lights-on time and ZT12 as the lights-off time), and the blood and lungs were collected.

### 2.3. Lung Morphometric Analysis

The completely cut-down left lung were fixed in 10% formalin and embedded in paraffin for making hematoxylin-eosin stained (HE) slides, morphometric analysis and further immunohistochemistry using slides. The HE slides were analyzed by optical microscope with a digital camera. The right lung tissues were frozen in liquid nitrogen immediately for further RNA extraction. Other organs were collected, weighted and cryopreserved for further analysis.

### 2.4. Blood and Tissue Biomarker Assays

After euthanasia, the blood was collected in EDTA anticoagulant tubes (Xinle Sci. & Tech. Co., Ltd., Shijiazhuang, China), centrifuged at 3000 rpm for 10 min at 4 °C. The EDTA-plasma was separated and collected for malondialdehyde (MDA) and glutathione (GSH) assay kit analyses. Thirty g each of lung tissue were ground in 1 mL PBS on ice, centrifuged at 5000 rpm for 15 min. The clarifying middle layer was carefully aspirated for MDA and 8-isoprostane assay kit analyses. Plasma and lung oxidative stress biomarkers were measured using enzymatic colorimetric assays.

### 2.5. Second-Generation RNA Sequencing for Gene Expression Analysis

Second-generation RNA sequencing (RNA-Seq) was used to analyze the genome-wide gene expression profiling in the lungs. RNA-Seq was analyzed by a commercial company (BGI, Beijing, China). We compared the transcriptomes in the lung of filtered group and unfiltered group by second-generation RNA sequencing. The gene expression level across all samples was evaluated by the bio-conductor package EDGER4. And the weakly expressed genes were filtered out when they had <1 read per million. Gene expression results are presented as the log_2_ ratio of data from the unfiltered air group versus the data from the filtered air group (log_2_ Ratio (unfiltered/filtered)). We sorted the lungs into four groups for RNA sequencing, the first time unfiltered mother (with normal pups) vs. first time filtered mother, the first unfiltered mother (with abnormal pups) vs. first time filtered mother, the second time unfiltered mother (with normal pups) vs. second time filtered mother, the unfiltered offspring (8 weeks) vs. filtered offspring (8 weeks).

### 2.6. Real-Time PCR for Verification of Selected Genes

The real time PCR (qPCR) was used for verifying the significantly up or down regulated genes which were given out by the RNA-Seq analysis as described above. We tested all 9 rhythm genes by qPCR (Supplementary Materials Table S1 showed the primer of genes). The analysis was performed on an Opticon 2 RT-PCR machine (Bio-Rad, Hercules, CA, USA) using SYBR Green mix (Tiangen, Beijing, China) with the following cycling program: 95 °C for 10 min, 40 cycles of 95 °C for 25 s, and 60 °C for 1 min. Glyceraldehyde 3-phostaphate dehydrogenase (GAPDH) was used as an internal control for normalization, and each sample was run in triplicate. The aliquot of cDNA was amplified with a pair of primers for each.

### 2.7. Statistical Analysis

SAS 9.1.3 for windows (SAS Institute Inc., Cary, NC, USA) was used to perform data analysis. The data were expressed as mean ± standard deviation (mean ± SD) in tabulate format. Pairwise comparison was conducted using Student’s *t*-test (two tailed) with equal or unequal variance depending on F-test results. *p*-value of less than 0.05 was considered statistically significant.

## 3. Results

### 3.1. The Pulmonary Inflammation of Lung Histology

**Figure 1:**
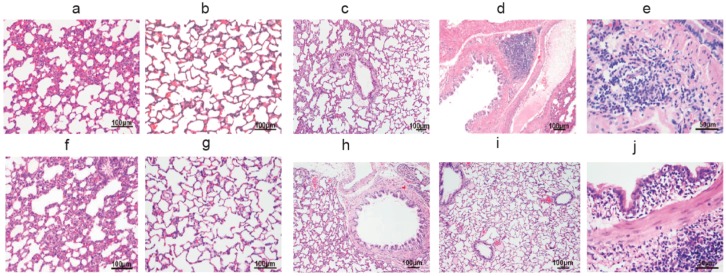
Lung histology (HE stain) of pregnant rats and 8-week-old male pups. (**a**) The alveolus of a pregnant rat exposed to unfiltered air; (**b**) the alveolus of a pregnant rat exposed to filtered air; (**c**) the bronchus of an unfiltered air exposed pregnant rat; (**d**) the bronchus of a filtered air exposed pregnant rat; (**e**) the mononuclear inflammatory infiltrates of an unfiltered air exposed pregnant rat; (**f**) The alveolus of 8-week-old rats exposed to unfiltered air; (**g**) the alveolus of 8-week-old male pups exposed to filtered air; (**h**) the bronchus of an unfiltered air exposed 8-week-old male pup; (**i**) the bronchus of an filtered air exposed 8-week-old male pup; (**j**) the mononuclear inflammatory infiltrates of an unfiltered air exposed 8-week-old male pup.

Both the lung sections of maternal and 8-week-old unfiltered air rats exhibited significant inflammation compared with the filtered air group. In pregnant rats, light microscopic examination in the unfiltered air group showed thickened alveolar septum, inflammatory cells in alveolar spaces and inflammation in the bronchus in comparison to filtered air exposed rats. The histologic sections at higher magnification show thickened alveolar septa and the presence of inflammatory cells in the airways of the unfiltered air groups compared to the lung tissues of rats in the filtered air chamber. Similar to that for the rat dams, lung histology showed both peribronchial and perivascular inflammation in these rats’ offspring (Figure 1).

### 3.2. The Oxidative Stress Factors in Plasma and Lung

**Figure 2:**
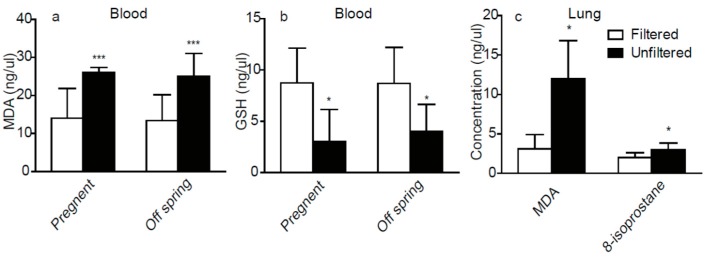
The oxidative stress factors in plasma and Lung. (**a**) Blood biomarkers, malondialdehyde (MDA), measured in pregnant rats and 8-week-old male pups; (**b**) Blood biomarkers, GSH, measured in pregnant rats and 8-week-old male pups; (**c**) Biomarkers of oxidative stress, malondialdehyde (MDA) and 8-isoprostane, in lungs of the 8 weeks old male pups. Significant difference between the two groups denoted as *p < 0.05, ***p < 0.001, *n* = 6 for each group.

As shown in Figure 2, the maternal and 8-week-old rats in the unfiltered air group showed significantly different plasma and lung tissue factors compared to the filtered air group. 

Accordingly, there were significant reductions of GSH and significant increases of MDA in plasma in the maternal and 8-week-old rats in the unfiltered air group. Furthermore, we observed significantly higher concentrations of MDA and 8-isoprostane in lung tissues in the unfiltered air group compared to the filtered air group of 8-week-old pups.

### 3.3. The Circadian Rhythm Genes in Lung

We overlapped all the RNA-Seq data of the four groups of lungs and picked out the same significantly expressed genes (Supplementary Materials Table S2). By using the DAVID bioinformatics software for functional annotation analyses, we identified several changed pathways. Our results showed that compared to the filtered air group, the circadian rhythm function was at the 4th position, just after the three inflammatory functions (Supplementary Materials Table S3). Thus, we further investigated the rhythm-related genes in PM exposed rats’ lungs.

**Figure 3:**
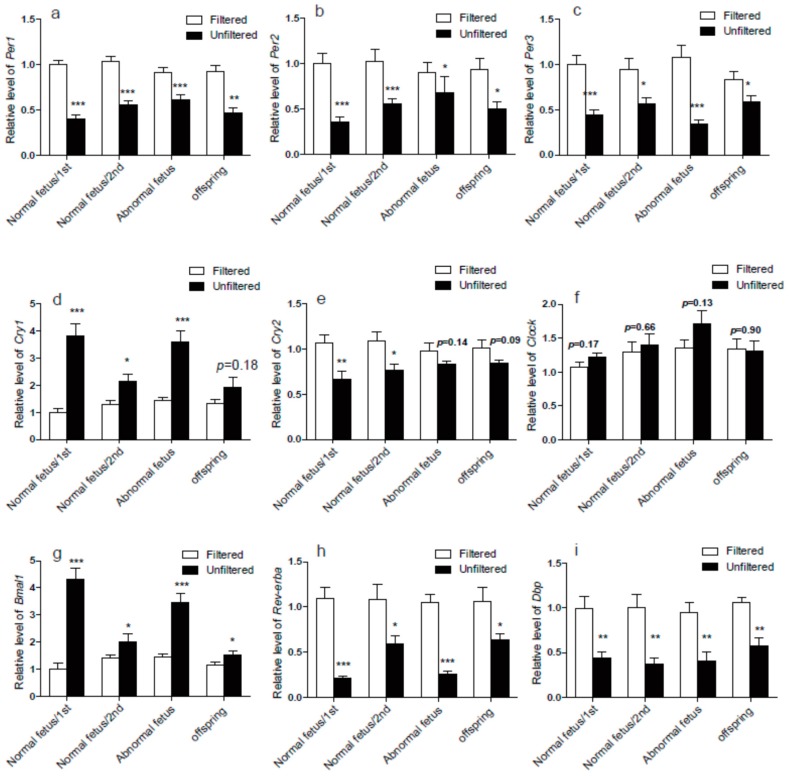
The circadian rhythm genes expression levels by real-time PCR. The *Per1* (**a**); *Per2* (**b**); *Per3* (**c**); *cry2* (**e**); *Rev-erbα* (**h**) and *Dbp* (**i**) expression levels decreased significantly in all groups after PM exposure. The *Cry1* (**d**) and *Bmal1* (**g**) expression levels were increased in all groups. The *Clock* (**f**) expression level did not change both in mothers and offspring. Significant difference between the two groups denoted as *p < 0.05, **p < 0.01, ***p < 0.001, *n* = 6 for each group.

The data of RNA-seq and real-time PCR showed that the mRNA expression level of the core rhythm genes of *Per1*, *Per2*, *Per3* decreased and *Cry1* and *Bmal1* increased significantly after PM exposure in both the gestational mothers and their 8-week-old offspring, but the *Cry2* expression level was decreased in the gestational mothers significantly, while it was only changed a little without meeting the requirement of |log_2_ ratio ≥ 0.5| (Supplementary Materials Table S4, Figure 3). In pups, it showed non-significantly decreased trends (Supplementary Materials Table S4, Figure 3). The other rhythm-related genes of *Rev-erbα* and *Dbp* also significantly decreased after PM exposure in both the gestational mother and the 8 weeks old offspring (Supplementary Materials Table S4, Figure 3). The expression level of *clock* did not change in both gestational mothers and 8 week old offspring (Supplementary Materials Table S4, Figure 3).

## 4. Discussion

In the present study, we used a set of exposure chambers to expose rats to real-life air particles. We then did a pathological assessment, biochemical analysis, and gene expression assays. We also examined molecular processes involved in the pathophysiological pathways by measuring RNA and protein biomarkers.

In both the maternal and the 8-week-old offspring rats, we observed pathological changes corresponding to perivascular and peribronchial inflammation in the lungs, as well as increased concentrations of MDA and decreased concentration of GSH in the blood. Furthermore, the analysis of lungs of 8-week-old offspring showed increased concentrations of MDA and 8-isoprostane. All these changes suggest oxidative stress in the lung and blood circulatory system, which are consistent with previous studies of the adverse health effects from ambient air particle exposure [20].

The bioinformatics analyses of RNA-Seq showed clearly that circadian rhythm-related functions and pathways were significantly altered right after the inflammation and stimulation. Previous studies have shown that ROS can affect the biological clock function. The fluctuation of the ROS level in the body can also change the redox buffer system within cells, thus changing the NADPH-dependent circadian gene Bmal1 transcription factor protein [21,22,23]. On the contrary, the circadian genes can also in turn regulate the ROS balance in biological systems, with overproduction of ROS and chronic oxidative stress responses [24]. Obviously, in our study, all the rats after PM exposure were in an oxidative stress situation indicated by the MDA, GSH and 8-isoprostane expression changes in blood and lung tissue. The circadian rhythm function in lungs was also changed. Thus, we have the proof to determine that the real-life PM exposure can lead to the unbalanced redox state as well as abnormal rhythm processes. However, the underlying molecular mechanisms of clock-controlled regulation of protective redox system and their roles in tissue-specific physiology and pathology still need to be studied in more depth.

The mechanisms of the main circadian clock involve intracellular auto-regulatory transcriptional loops of specific genes called clock genes (CCGs), including *Per1*, *Per2*, *Per3*, *Clock*, *Bmal1*, *Cry1*, and *Cry2* [25]. *Rev-erbα* was also an integrator of circadian rhythms [26]. Generally, the circadian clock mechanism consists of cell-autonomous transcription-translation feedback loops (TTFL) that drive rhythmic, the 24-h expression patterns of core clock components [5]. There are two loops, the positive feedback loop of Bmal1/Clock and the negative loop of period (*Per1-3*) and cryptochrome (*Cry1-2*). The Bmal1/Clock transcriptional activator complex was upstream of the Period (*Per1-3*) and Cryptochrome (*Cry1-2*) genes, while *Per1-3* and *Cry1-2* repress Bmal1/Clock to form an autoregulatory transcriptional feedback loop [27,28]. In the present study, as shown in Figure 3, compared to the unfiltered group, the core rhythm genes of *Per1*, *Per2*, *Per3* decreased and *Bmal1* increased after PM exposure in both the gestational mother and the 8-week-old offspring. It is probably because that decreased *Per1-3* resulted in the reduced repression of *Bmal1*. Meanwhile, the increased expression level of *Bmal1* activated the expression of *Cry1* in gestational mother. Moreover, the Bmal1/Clock complex can activate the *Rev-erbα* expression and the over-expression of *Rev-erbα* also represses *Bmal1* transcription [29]. In both the gestational mother and the 8-week-old offspring, the high level down-regulation of *Rev-erbα* promoted the increase of *Bmal1*. In addition, the *Rora* as a component of the mammalian circadian, was involved in normal *Bmal1* expression [30]. Casein kinase I can partially activate the transcription factor BMAL1 [31], but the PM exposure did not influence the expression of the *Rora* and Casein kinase I in our RNA-seq data. Regarding the different expression of clock genes in the rat dams and pups, we can only make the ambiguous inference that the prenatal exposure to PM maybe not have an effect on the offspring, but this should be confirmed in the future.

Circadian genes have also been determined to have a relationship with immunity and inflammation. An increased neutrophil response is associated with many inflammatory diseases presented in significant circadian rhythm cycle [32,33]. In the present study, we found many inflammatory and immunologic genes were up-regulated in the lung RNA-Seq results. Among them, Il6, Il10, Cxcl14, Ccl2, Ccl11, Ccl20, Ifnk, Tnf, Ctf, Il1b, lifr and Tnfsf10 refer to the Cytokine-cytokine receptor interaction function; Cxcl1, Cxcl2, Cxcl6, Cxcl14, Ccl2, Ccl9, Ccl11, Ccl20, Gng10 and Adcy9 were involved in the chemokine signaling pathway; Cxcl1, Cxcl2, Ccl2, Ccl20, Il1b, Il6 and Tnf play roles in the TNF signaling pathway. Some in vivo studies have observed that the inflammatory stimulation could regulate the rhythm genes in SCN and peripheral tissues, such as *Per1*, *Per2* and *Bmal1* [34,35,36]. Environmental stress induced lung inflammation in lung epithelial cell-specific BMAL1 knockout animals displayed an increased proinflammatory cytokine gene expression (*ccl1/mcp-1* and *cxcl1/kc*) and cytokine release (CCL1/MCP-1and CXCL1/KC) [37]. Moreover, BMAL1 knockout animals displayed up-regulation of the TNFα, and IL6, suggesting that molecular clock disruption can lead directly to inflammatory responses [38]. The nuclear heme receptor REV-ERBα attenuates the activation of IL6 expression [39]. REV-ERBα also binds to nuclear factor-κB (NF-κB), which can activate NF-κB-dependent transcription [40]. In the current study we found that gestational PM exposure led to altered levels of clock gene expression in rat lungs. However, whether this can transfer to the next generation needs to be studied in the future. Based on the inextricable association between inflammation and clock genes, we speculate that the alteration of the level of peripheral clock genes expression after chronic exposure to particulate matter maybe another important pathway for explaining the feedback of ambient particle exposure in addition to oxidative stress and inflammation.

## 5. Conclusions

In conclusion, our study showed that chronic exposure to particulate matter caused alterations of clock genes expression in rats’ lung tissue, as well as increased lung inflammation. Previous studies demonstrated that the molecular clock genes could regulate the pulmonary inflammation and injurious responses. Our results highlighted the association between PM exposure and clock genes, which implicated that the alteration of clock genes expression may have a great effect on PM-mediated lung inflammation. It is well known that exposure to atmospheric particulates can cause cardiovascular disease. A better understanding of the relationship between clock genes and pulmonary inflammation caused by PM can help us to find ways for effective prevention and treatment. In the future, much more precise experiments need to be conducted, for example, what is the time point when circadian rhythm genes begin to change, whether the changes in circadian genes expression occur earlier and if the trend will be maintained, etc.

## Supplementary Materials

The following are available online at www.mdpi.com/1660-4601/14/1/90/s1, Table S1: The primer for real-time PCR, Table S2: Overlap genes of 4 groups data of RNA-Seq, Table S3: DAVID bioinformatics for functional annotation, Table S4: The circadian gene expression level, Table S5: The top 50 up-regulated genes and top 50 down-regulated genes of the first set of experiments (Mothers with normal fetus), Table S6: The top 50 up-regulated genes and top 50 down-regulated genes of the second set of experiments (Mothers with normal fetus), Table S7: The top 50 up-regulated genes and top 50 down-regulated genes of the set of experiments (Mothers with abnormal fetus), Table S8: The top 50 up-regulated genes and top 50 down-regulated genes of offspring.

## Abbreviations

The following abbreviations are used in this manuscript:

COPD	Chronic Obstructive Pulmonary Disease
GSH	Glutathione
GAPDH	Glyceraldehyde 3-phostaphate Dehydrogenase
HE stained	Hematoxylin-Eosin Stained
HEPA	High-Efficiency Particulate Air
MDA	Malondialdehyde
NF-κB	Nuclear Factor-κB
PM_2.5_	Particulate Matter with an aerodynamic diameter less than 2.5 μm
qPCR	Real-Time PCR
ROS	Reactive Oxygen Species
RNA-Seq	Second Generation RNA Sequencing
SCN	Suprachiasmatic Nucleus
TTFL	Cell-Autonomous Transcription-Translation Feedback Loops
