# Supplementary material for: Transcriptome Profiling of the Lungs Reveals Molecular Clock Genes Expression Changes after Chronic Exposure to Ambient Air Particles
Source: Int J Environ Res Public Health. 2017 Jan 18;14(1):90. doi: 10.3390/ijerph14010090 (PMC5295340; doi:10.3390/ijerph14010090)
Supplement: Supplementary file 1 [file ijerph-14-00090-s001.pdf]

# Supplementary Materials: Transcriptome Profiling of the Lungs Reveals Molecular Clock Genes Expression Changes after Chronic Exposure to Ambient Air Particles

**Pengcheng Song, Zhigang Li, Xiaoqian Li, Lixin Yang, Lulu Zhang, Nannan Li, Chen Guo, Shuyu Lu and Yongjie Wei**

**Table S1.** The primers for real-time PCR.

| Gene Name | Forward (5'—3') | Reverse (5'—3') |
|---|---|---|
| *Per1* | CAGCAGTGGAGTCTGGAGGA | TAGGAGCTCTGAGAAGCGGG |
| *Per2* | AGCCCTGCAGCATGGAAGTA | ACGTCATGAGGAGCCAGGAA |
| *Per3* | TGTGTTCAAGGGTCCACTGC | GGTGCTGGCAACTTCTTTCG |
| *Bmal1* | CCAAGAAAGTATGGACACAGACAAA | GCATTCTTGATCCTTCCTTGGT |
| *Clock* | TTGCTCCACGGGAATCCTT | GGAGGGAAAGTGCTCTGTTGTAG |
| *Cry1* | CTGGCGTGGAAGTCATCGT | CTGTCCGCCATTGAGTTCTATG |
| *Cry2* | TGTCCCTTCCTGTGTGGAAGA | GCTCCCAGCTTGGCTTGA |
| *REV-ERBα* | GGGCACAAGCAACATTACCA | CACGTCCCCACACACCTTAC |
| *Dbp* | AAGGAGCGCAAGGCAACTCT | AGATGTCAAGCCTGCGCGGT |
| *Gapdh* | CCCTTAAGAGGGATGCTGCC | TACGGCCAAATCCGTTCACA |

**Table S2.** Overlap genes of 4 groups data of RNA-Seq.

| Adcy9 | Cxcl14 | Per1 | A2m |
|---|---|---|---|
| Arntl | Cxcl2 | Per2 | Alas1 |
| Ccl11 | Cxcl6 | Per3 | Alox15 |
| Ccl2 | Gng10 | Tnf | Ddit4 |
| Ccl20 | Ifnk | Tnfsf10 | Efnb2 |
| Ccl9 | Il10 | Hif3a | Epas1 |
| Cry1 | Il1b | Trim16 | Fabp4 |
| Ctf1 | Il6 | Prl8a2 | Tef |
| Cxcl1 | Lifr | Prl4a1 | |

**Table S3.** DAVID bioinformatics for functional annotation.

| Term | Count | % | *p*-Value |
|---|---|---|---|
| Cytokine-cytokine receptor interaction | 12 | 34.2857143 | 1.27E-10 |
| Chemokine signaling pathway | 10 | 28.5714286 | 8.32E-09 |
| TNF signaling pathway | 7 | 20 | 2.31E-06 |
| Circadian rhythm | 5 | 14.2857143 | 4.03E-06 |
| Herpes simplex infection | 8 | 22.8571429 | 1.05E-05 |
| Rheumatoid arthritis | 6 | 17.1428571 | 1.60E-05 |
| Legionellosis | 5 | 14.2857143 | 5.41E-05 |
| Malaria | 5 | 14.2857143 | 6.20E-05 |
| Pertussis | 5 | 14.2857143 | 1.43E-04 |
| African trypanosomiasis | 4 | 11.4285714 | 3.62E-04 |
| Circadian entrainment | 5 | 14.2857143 | 4.28E-04 |
| Chagas disease (American trypanosomiasis) | 5 | 14.2857143 | 6.21E-04 |
| NOD-like receptor signaling pathway | 4 | 11.4285714 | 0.001137577 |
| Jak-STAT signaling pathway | 5 | 14.2857143 | 0.001482411 |
| Inflammatory bowel disease (IBD) | 4 | 11.4285714 | 0.001752567 |

| | | | |
|---|---|---|---|
| Influenza A | 5 | 14.2857143 | 0.003511071 |
| Salmonella infection | 4 | 11.4285714 | 0.00352409 |
| Asthma | 3 | 8.57142857 | 0.005493904 |
| Amoebiasis | 4 | 11.4285714 | 0.008130768 |
| Graft-versus-host disease | 3 | 8.57142857 | 0.024937127 |
| Leishmaniasis | 3 | 8.57142857 | 0.029309668 |
| Tuberculosis | 4 | 11.4285714 | 0.029619359 |
| Hematopoietic cell lineage | 3 | 8.57142857 | 0.036396184 |
| Toll-like receptor signaling pathway | 3 | 8.57142857 | 0.050428394 |
| Measles | 3 | 8.57142857 | 0.087620423 |
| FoxO signaling pathway | 3 | 8.57142857 | 0.088747392 |

**Table S4.** The circadian gene expression level.

| | **$Log_2$Ratio (Unfiltered/filtered)/Mother** | | | | | | **$Log_2$Ratio (Unfiltered/Filtered)/ Offspring** | |
|---|---|---|---|---|---|---|---|---|
| | **Normal Fetus/1st** | ***p*-Value** | **Normal Fetus/2nd** | ***p*-Value** | **Abnormal Fetus** | ***p*-Value** | **8 Weeks** | ***p*-Value** |
| *Per1* | −1.945 | 0.0000 | −1.864 | 0.0000 | −1.816 | 0.0000 | −1.424 | 0.0000 |
| *Per2* | −0.381 | 0.0177 | −3.226 | 0.0000 | −1.678 | 0.0000 | −1.146 | 0.0000 |
| *Per3* | −2.279 | 0.0000 | −1.775 | 0.0000 | −3.448 | 0.0000 | −1.221 | 0.0000 |
| *Cry1* | 2.412 | 0.0000 | 1.071 | 0.0002 | 2.124 | 0.0000 | 0.610 | 0.0502 |
| *Cry2* | −0.287 | 0.0642 | −0.091 | 0.0000 | −0.073 | 0.0000 | 0.049 | 0.7750 |
| *Clock* | 0.036 | 0.9369 | −0.396 | 0.4581 | 0.287 | 0.5256 | −0.887 | 0.1147 |
| *Bmal1* | 2.144 | 0.0000 | 1.966 | 0.0000 | 2.466 | 0.0000 | 0.695 | 0.0293 |
| *Rev-erbα* | −5.659 | 0.0000 | −2.877 | 0.0000 | −5.362 | 0.0000 | −0.359 | 0.0001 |
| *Dbp* | −3.551 | 0.0000 | −3.870 | 0.0000 | −3.492 | 0.0000 | −.318 | 0.0204 |

$|\log_2 \text{Ratio}| \geq 0.5$ and $p$ value < 0.05 are significant.

**Table S5.** The top 50 up-regulated genes and top 50 down-regulated genes of the first set of experiments (Mothers with normal fetus).

| **Up-Regulated** | | **Down-Regulated** | |
|---|---|---|---|
| **Gene Name** | **$Log_2$ Ratio (Unfiltered/Filtered)** | **Gene Name** | **$Log_2$ Ratio (Unfiltered/Filtered)** |
| LOC688228 | 17.10880447 | Hbe1 | −14.54798886 |
| Myh7 | 15.39361452 | Atp6v0a4 | −11.87580404 |
| Mb | 15.06807234 | *Rev-erbα* | −5.659 |
| Sln | 14.96594354 | Setbp1 | −4.548619992 |
| RatNP-3b | 13.60572642 | Gli3 | −4.487219447 |
| Cxcl6 | 13.47712177 | LOC100361038 | −4.133582493 |
| Smpx | 13.13154909 | Krt75 | −4.133582493 |
| LOC688228 | 13.02491448 | RGD1566296 | −3.821638486 |
| Nppa | 12.98516145 | LOC691925 | −3.578367335 |
| Myoz2 | 12.89710001 | Vsig4 | −3.548619992 |
| Pthlh | 12.73528145 | *Dbp* | −3.551 |
| Tnfrsf13c | 12.60433509 | Nr1d2 | −3.51281927 |
| Mybpc3 | 12.30320783 | Tbx18 | −3.332891301 |
| Jsrp1 | 11.90578723 | LOC100359743 | −3.285585586 |
| Ckm | 11.78715365 | Rprml | −3.165291352 |
| Lmod2 | 11.69406814 | Pigzl1 | −3.133582493 |
| Krt77 | 11.6684463 | Slc16a12 | −3.092940508 |
| Lrrc10 | 11.63530344 | Cyp2b2 | −3.051120332 |
| Adam28 | 11.57331715 | RGD1563615 | −2.771012413 |
| Silv | 11.28274372 | Gm672 | −2.718544993 |
| Grem1 | 10.97864428 | Vdr | −2.718544993 |

| | | | |
|---|---|---|---|
| LOC681849 | 10.81805593 | Slc38a7 | −2.718544993 |
| Tmem182 | 10.68144006 | Abcb7 | −2.718544993 |
| Vsx1 | 10.62604547 | Rasd2 | −2.632036 |
| Vwa5b1 | 10.46217143 | RGD1565166 | −2.621188618 |
| Xirp2 | 9.463552884 | Hyal1 | −2.61900932 |
| Sspo | 8.421839333 | Tlr8 | −2.578367335 |
| Plunc | 8.227154877 | RGD1566226 | −2.478230664 |
| Il6 | 8.14689435 | Msr1 | −2.42308911 |
| Il10 | 7.825518061 | RGD1359290 | −2.402769125 |
| Clca3 | 7.074163974 | Wfs1 | −2.401062803 |
| Gp2 | 6.330963258 | Dpep3 | −2.389922246 |
| Scgb3a1 | 5.931160272 | Fras1 | −2.363588098 |
| Ccl20 | 5.428659932 | Fam46b | −2.339524393 |
| Itln1 | 4.514389806 | Mtcp1 | −2.317294446 |
| Tnnt2 | 4.506228485 | Tmem37 | −2.285585586 |
| Actn2 | 4.495774128 | Upk3b | −2.285585586 |
| C9 | 4.30312905 | Kcnn3 | −2.285585586 |
| Cd177 | 3.828900429 | Per3 | −2.2786992 |
| B3gnt5 | 3.780503604 | LOC690492 | −2.204665591 |
| Ccl19 | 3.746000757 | Tmem86b | −2.200696688 |
| RGD1563047 | 3.730239381 | Slc10a6 | −2.186049912 |
| Tnnc1 | 3.718166549 | Arrb1 | −2.176651214 |
| Cxcl13 | 3.708767851 | Lrrn4 | −2.145860822 |
| Cdh26 | 3.62130501 | Ablim2 | −2.133582493 |
| Glycam1 | 3.532768335 | RGD1565536 | −2.089188373 |
| Ccr6 | 3.445733445 | Ficd | −2.079134709 |
| Fcer2 | 3.428659932 | Susd2 | −2.005316643 |
| Scin | 3.428659932 | Mfsd2 | −1.996078969 |
| Cox6a2 | 3.313182714 | Abca17 | −1.993404835 |

**Table S6.** The top 50 up-regulated genes and top 50 down-regulated genes of the second set of experiments (Mothers with normal fetus).

| Up-Regulated | | Down-Regulated | |
|---|---|---|---|
| **Gene Name** | **$Log_2$ Ratio (Unfiltered/Filtered)** | **Gene Name** | **$Log_2$ Ratio (Unfiltered/Filtered)** |
| Zpbp2 | 11.94984935 | Tpbpa | −14.72823222 |
| Myh7 | 9.846311265 | Hbe1 | −14.54798886 |
| Cxcl6 | 9.846311265 | Cyp2b2 | −13.36681948 |
| Il10 | 8.940621387 | Prl4a1 | −13.24075465 |
| Il6 | 8.261997677 | Ceacam11 | −12.80285399 |
| Plunc | 6.268309593 | LOC100360737 | −12.39417223 |
| Ccl20 | 3.321370837 | LOC100361038 | −11.75559621 |
| Scgb3a1 | 2.800311388 | Cgm4 | −11.43951871 |
| Adh7 | 2.695766351 | Gnao1 | −11.21466135 |
| Retnla | 2.434504875 | Prl8a5 | −4.892948284 |
| Slc26a4 | 2.373838256 | RT1-T24-1 | −4.372116121 |
| LOC100359517 | 2.229448347 | *Dbp* | −3.870 |
| Ly6al | 2.223854331 | LOC100362483 | −3.769119697 |
| Trim16 | 2.215576173 | RGD1566401 | −3.492410355 |
| F13a1 | 2.105642146 | Per2 | −3.225523623 |
| Chodl | 2.09004529 | Sptb | −3.096481678 |
| Cfi | 2.029767279 | Pilra | −3.077372855 |
| LOC687234 | 1.974568073 | Art4 | −2.977837182 |
| Arntl | 1.965890182 | *Rev-erbα* | −2.877 |
| Ngp | 1.958800757 | Vil1 | −2.736079436 |
| LOC100361788 | 1.942859213 | C6 | −2.736079436 |
| Tnf | 1.888458204 | Slc4a1 | −2.7346862 |

| | | | |
|---|---|---|---|
| Ccl2 | 1.883305716 | Tor3a | −2.655909087 |
| Dmbt1 | 1.87936629 | Cacna1h | −2.585519759 |
| Abhd3 | 1.857714632 | Wfikkn1 | −2.585519759 |
| Tekt3 | 1.851885553 | Spred2 | −2.548993883 |
| Rnf11 | 1.848518702 | Fndc3b | −2.457363408 |
| Pcmtd2 | 1.799734271 | RT1-S2 | −2.36640247 |
| Ptma | 1.799734271 | RT1-Cl | −2.346053824 |
| Atg4c | 1.799144091 | RGD1565166 | −2.343813863 |
| Ap3s1 | 1.772934212 | Tbxa2r | −2.307985783 |
| LOC362795 | 1.764977488 | Plekha6 | −2.293339008 |
| Ly6b | 1.712856923 | Dos | −2.233218015 |
| LOC100365063 | 1.709441288 | Atp6v0a4 | −2.206106169 |
| Kcne3 | 1.706034687 | Ddit4 | −2.129510479 |
| Crp | 1.694080158 | Hyal1 | −2.12539437 |
| Il1b | 1.686990734 | Vsig4 | −2.111588571 |
| S100a8 | 1.68657718 | Mfap3 | −2.06005827 |
| Rfc4 | 1.660459483 | LOC500300 | −2.041199243 |
| RGD1563325 | 1.620931118 | Myh7b | −2.036181168 |
| RGD1308612 | 1.605163803 | RGD1308124 | −2.000557258 |
| Aurkb | 1.597245588 | Gabrp | −2.000557258 |
| Lcn2 | 1.556702314 | RGD1562037 | −1.943711398 |
| Yipf4 | 1.549995212 | LOC100363476 | −1.942736604 |
| Naip2 | 1.540488126 | Cd52 | −1.936017006 |
| Gng10 | 1.535495642 | Rasd2 | −1.931970173 |
| Zfp758 | 1.502918206 | Wfs1 | −1.870921978 |
| Pon1 | 1.497220672 | Per1 | −1.864076437 |
| Mrpl1 | 1.497220672 | Hba-a2 | −1.836132606 |
| MGC105649 | 1.491674747 | Cdc25b | −1.828376283 |

**Table S7.** The top 50 up-regulated genes and top 50 down-regulated genes of the set of experiments (Mothers with abnormal fetus).

| **Up-Regulated** | | **Down-Regulated** | |
|---|---|---|---|
| **Gene Name** | **Log$_2$ Ratio (Unfiltered/Filtered)** | **Gene Name** | **Log$_2$ Ratio (Unfiltered/Filtered)** |
| Ctsq | 13.40182003 | Hbe1 | −14.54798886 |
| Cxcl6 | 12.8239556 | Cyp2b2 | −13.36681948 |
| LOC100360968 | 12.50664804 | Atp6v0a4 | −11.87580404 |
| Myh7 | 11.01092757 | LOC100361038 | −11.75559621 |
| Krt77 | 10.96523945 | LOC367975 | −11.60012236 |
| Il10 | 10.12231121 | Adcy9 | −11.27521286 |
| Il6 | 9.121759402 | Slc39a5 | −10.84557757 |
| Plunc | 7.856697612 | LOC100359544 | −10.73844375 |
| Ccl20 | 5.534769515 | *Rev-erbα* | −5.362 |
| Prl3d4 | 3.918098157 | RGD620382 | −4.632648628 |
| Clca3 | 3.797803923 | RT1-T24-1 | −4.512354395 |
| Ifnk | 3.646796135 | Prss35 | −4.236719952 |
| Scgb3a1 | 3.490460621 | Mlc1 | −3.689232157 |
| H19 | 3.426245061 | *Dbp* | −3.492 |
| Prl3b1 | 3.368759566 | Per3 | −3.448224057 |
| Ctsr | 3.353599759 | Zbtb16 | −3.323776686 |
| Prl8a7 | 3.26994183 | Cd300lg | −3.07625528 |
| Ly6al | 3.259135075 | Pilra | −3.047686128 |
| Trim16 | 3.224201285 | Nsg2 | −3.047686128 |
| Prl8a4 | 3.084456543 | Nr1d2 | −2.981560869 |
| Prl2a1 | 2.974681685 | Hyal1 | −2.966072362 |
| LOC100360737 | 2.877657231 | RGD1561778 | −2.796147361 |
| A2m | 2.859204468 | Rnasel | −2.796147361 |

| | | | |
|---|---|---|---|
| Cxcl2 | 2.818562483 | RGD1305469 | −2.403829938 |
| Prl4a1 | 2.783797065 | Hipk4 | −2.373456289 |
| RGD1562420 | 2.782791352 | Hgd | −2.358026248 |
| Prl8a2 | 2.766095063 | Vsig4 | −2.351362518 |
| Ceacam11 | 2.766095063 | RGD1566401 | −2.310720534 |
| Retnla | 2.686137272 | Lef1 | −2.225831636 |
| Tpbpa | 2.684489865 | Bmp3 | −2.21118486 |
| Prl8a9 | 2.620016804 | Usp2 | −2.198245804 |
| Arntl | 2.466109521 | Ddit4 | −2.185684488 |
| Slc26a4 | 2.426245061 | Atp2c2 | −2.143120585 |
| Car3 | 2.421491531 | Slc4a1 | −2.097316895 |
| LOC100362680 | 2.333135656 | LOC100364166 | −2.083949672 |
| Ctsql2 | 2.313770331 | Dos | −2.083949672 |
| Sh3rf2 | 2.267547315 | RGD1305090 | −2.024416348 |
| RGD1564827 | 2.212841422 | Pusl1 | −2.01048751 |
| Ly6b | 2.212210874 | LOC691259 | −1.988792439 |
| Cry1 | 2.123682291 | Spred2 | −1.988792439 |
| Tnf | 2.011207562 | RGD1566226 | −1.988792439 |
| LOC687234 | 2.011207561 | Ccdc129 | −1.988792439 |
| LOC684097 | 1.978040697 | Dpep3 | −1.955625575 |
| Cxcl1 | 1.968979326 | LOC641523 | −1.944849091 |
| Ccl2 | 1.886098931 | Rnf24 | −1.93220891 |
| LOC683983 | 1.865356695 | LOC100360801 | −1.911624578 |
| Igf2 | 1.811438049 | Kcnj15 | −1.830094693 |
| Prl8a5 | 1.799703456 | Coro6 | −1.824372543 |
| Ap3s1 | 1.770199462 | Per1 | −1.81601118 |
| Cd177 | 1.727414595 | Cables1 | −1.796147361 |

**Table S8.** The top 50 up-regulated genes and top 50 down-regulated genes of offspring.

| Up-Regulated | | Down-Regulated | |
|---|---|---|---|
| **Gene Name** | **$Log_2$ Ratio (Unfiltered/Filtered)** | **Gene Name** | **$Log_2$ Ratio (Unfiltered/Filtered)** |
| LOC691925 | 14.42220984 | Afp | −12.69024474 |
| LOC690689 | 12.53245989 | Marveld2 | −12.64138316 |
| Ctrb1 | 12.2808846 | Kpna5 | −11.34108037 |
| Cav3 | 11.99075059 | LOC100359743 | −5.781532828 |
| Il6 | 3.960866699 | Kcnj15 | −5.190172556 |
| LOC363827 | 3.699493041 | Zbtb41 | −4.420785484 |
| LOC500375 | 3.562208091 | Shisa2 | −4.420785484 |
| Nuf2 | 2.992842445 | Asb7 | −4.28328196 |
| LOC641523 | 2.846001057 | Ctsq | −4.28328196 |
| Gabrp | 2.726702128 | LOC171573 | −4.246756084 |
| Defb5 | 2.444638494 | Slc36a1 | −4.209281379 |
| Slc5a5 | 2.404295607 | Dusp4 | −4.131278867 |
| LOC681420 | 2.311664629 | Psg29 | −4.131278867 |
| LOC100361706 | 2.240279996 | Prl3d4 | −4.076831083 |
| RGD1564209 | 2.185487523 | Prl3b1 | −4.031743193 |
| Ccl20 | 2.074270044 | Prl8a9 | −3.983721678 |
| Slc7a11 | 2.038646135 | Ctsr | −3.952308726 |
| Tmc5 | 2.014398588 | Ankrd44 | −3.938633789 |
| Osbpl8 | 2.012650926 | Slc7a2 | −3.819334861 |
| Il10 | 1.986861908 | H19 | −3.800557654 |
| Rgs4 | 1.98054318 | Prl8a4 | −3.611607826 |
| Pilra | 1.974515797 | Cyp2b2 | −3.605210055 |
| Tnf | 1.932774392 | LOC300314 | −3.484915822 |
| Cxcl14 | 1.932774392 | Sema3d | −3.420785484 |
| Exd1 | 1.926171405 | LOC100362680 | −3.420785484 |
| LOC690276 | 1.892795268 | Dcbld2 | −3.420785484 |

| | | | |
|---|---|---|---|
| LOC366772 | 1.838100416 | Rbm20 | −3.330587675 |
| RGD1564859 | 1.83511274 | Ctsql2 | −3.296956897 |
| Pon1 | 1.830841249 | Baat | −3.28328196 |
| Igh-1a | 1.823174946 | LOC100361884 | −3.209281379 |
| LOC499742 | 1.775611729 | Pde7b | −3.131278867 |
| Hp | 1.764471171 | Upk3b | −3.131278867 |
| Pcp4l1 | 1.761112159 | Gpr77 | −3.076831083 |
| RGD1565236 | 1.746465383 | Abca8 | −3.005747985 |
| LOC100363106 | 1.740991407 | Zbtb6 | −2.996977775 |
| Vom2r52 | 1.739085853 | Gpr137b | −2.899953321 |
| Mfap4 | 1.729343664 | RGD1560666 | −2.899953321 |
| Pcdh17 | 1.704908737 | Glt25d2 | −2.899953321 |
| Il1b | 1.677284916 | Ccdc6 | −2.899953321 |
| LOC303448 | 1.663136999 | Tmtc2 | −2.887353284 |
| Hmox3 | 1.641310637 | Fzd4 | −2.835822983 |
| Thnsl2 | 1.623608635 | Dlk1 | −2.819334861 |
| LOC100363076 | 1.606929894 | RGD1564827 | −2.804628362 |
| RGD1310371 | 1.581173369 | Lifr | −2.768708788 |
| Mki67 | 1.578379381 | Zbtb33 | −2.768708788 |
| Pard3b | 1.575911893 | Cyp26b1 | −2.727446822 |
| LOC100361520 | 1.568611358 | Fras1 | −2.711375612 |
| Tcf19 | 1.567025107 | Prl8a5 | −2.703857643 |
| Tmem138 | 1.535071961 | Serpina3n | −2.687178902 |
| Ctsw | 1.524072962 | Pstpip2 | −2.68381989 |

© 2017 by the authors. Submitted for possible open access publication under the terms and conditions of the Creative Commons Attribution (CC BY) license (http://creativecommons.org/licenses/by/4.0/).
